# Treatment of an infected nonunion with additional fresh fracture of the femur with a silver-coated intramedullary nail: A case report

**DOI:** 10.1016/j.tcr.2022.100641

**Published:** 2022-03-23

**Authors:** Volker Alt

**Affiliations:** Department of Trauma Surgery, University Hsopital Regensburg, Germany

**Keywords:** Silver, Coating, Infection, Fracture-related infection, FRI, Non-union, Fracture

## Abstract

Infected non-unions of the femur can be difficult to treat with a high risk of reinfection and persisting nonunion. Here we demonstrate a case of a 66-year-old female with a chronic infected nonunion of the left femur. The patient fell during temporary external fixation of the infected nonunion and sustained an additional proximal femur shaft fracture. The case was successfully managed with a low-amount silver coating of a long proximal femur nail and an additional augmentation locking plate along with local and systemic antibiotics. After a follow-up of 26 months, both the fracture and also the infected nonunion healed completely without recurrence of infection and without any signs of adverse events in response to the silver coating. In conclusion, silver coated fracture fixation devices can be helpful in difficult to treat infection cases without adverse events.

## Introduction

The treatment of infected non-unions of long bones can be difficult. The overall goal for this entity is infection control with a subsequent successful bone healing without recurrence of infection. Antimicrobial coatings of implants has been shown to the successful and without any severe complications in the management of complex infections in trauma, periprosthetic joint infections and orthopaedic oncology [1]. A gentamicin coated nail (ETN Protect©, DePuy Synthes, Bettlach, Schweiz) has been clinically evaluated with positive outcome for open fractures, non-unions and fracture-related infections over the last years [2], [3]. Potential risks in the use of gentamicin include antibiotic resistance of the infection causing microorganism and the induction of further resistance against gentamicin [1]. Silver is a viable alternative for antimicrobial coating of implants as it has a broad range of action against gram-positive and gram-negative bacteria as well as against fungi without any clinically relevant severe resistance [4]. Silver has been reported with different technologies for the coating of megaprosthesis prostheses [1], [5] but not for coating of intramedullary nails.

To our best knowledge, we here report the first case on the use of a silver-coated intramedullary nail. In this case, an infected non-union of the femur with an additional fresh fracture of the proximal shaft area was managed with a gamma nail with a low-amount silver coating.

## Clinical case

**Fig. 1:**
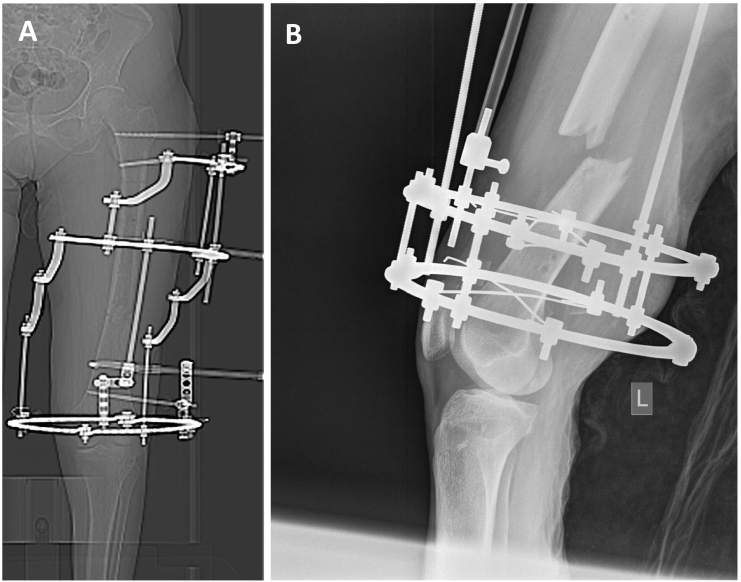
CT-overview scan (A) and lateral X-ray (B) of the distal left femur with external hybrod fixation and without any signs of callus formation four months after the initial trauma.

A 66-year-old female sustained a polytrauma accident with a Gustilo-Anderson type I open distal femur shaft fracture. The open femur fracture was initially treated with external fixation that was then changed to an intramedullary nail a few days later. The patient developed an acute fracture-related infection of the nail, which was then removed with a debridement of the infected fracture site and a hybrid fixateur with monolateral half pin at the proximal and ring fixator construct at the distal fragment was placed. Four months after initial trauma, the patient presented a persisting draining sinus at the lateral aspect of the fracture site. X-rays showed no signs of callus formation and the case was considered as chronically infected non-union of the distal femur (Fig. 1). A 2-stage treatment protocol was planned with removal of the hybrid fixateur, debridement of the nonunion site, implantation of a local antibiotic carrier system at stage 1 followed by re-debridement, removal of the antibiotic carrier and re-osteosynthesis with a silver coated gamma nail at stage 2. Informed consent from the patient was received for the general aspects of the 2-stage treatment and specifically for the use of a silver-coated implant as well.

**Fig. 2:**
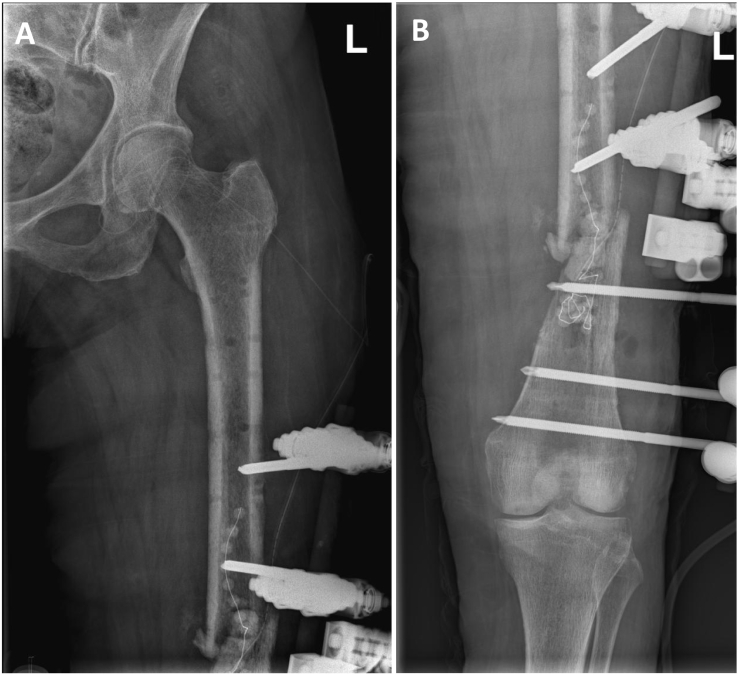
Postoperative X-rays after removal of the external hybrid fixation, debridement of the infected non-union site, placement of antibiotic beads and re-osteosynthesis by external fixation.

For stage 1, the hybrid fixateur with all indwelling pins was removed and pin tracks were debrided with a curette and cleaned. The infected non-union site was accessed via a lateral approach and thoroughly debrided resulting in a 3 cm long semi-circumferential defect of the dorsal femur around the non-union site. The intramedullary canal was also debrided by intramedullary reaming and gentamicin-loaded antibiotic beads (Septopal®, ZimmerBiomet, Berlin, Germany) were placed intro the intramedullary canal. The 3 cm semi-circumferential defect of the dorsal femur was filled with a vancomycin and gentamicin-loaded PMMA spacer. The femur was stabilized by a monolateral fixateur and the primary wound closure was performed (Fig. 2). Methicillin-susceptible *Staphylococcus aureus* and *Staphylococcus epidermidis* were found along with *E. coli* from intraoperative tissue specimens. Systemic antibiotic therapy with meropenem (3x1g i.v.) and vancomicin (1 g i.v.) was started.

**Fig. 3:**
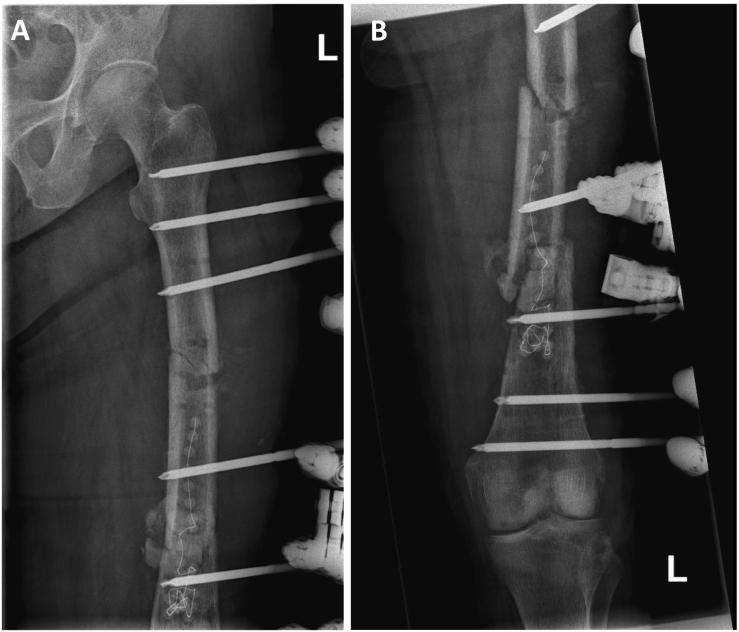
X-rays of the proximal (A) and distal femur (B) after fall with additional fresh fracture of the proximal femoral shaft and placement of additional pins into the proximal femur.

The patient fell two weeks after the first revision on her left site and sustained an additional fresh fracture of the proximal femur shaft. The patient was taken to the OR on the same day and close reduction of the fracture was performed together with extension of the monolateral fixateur to the proximal part of the femur (Fig. 3).

**Fig. 4:**
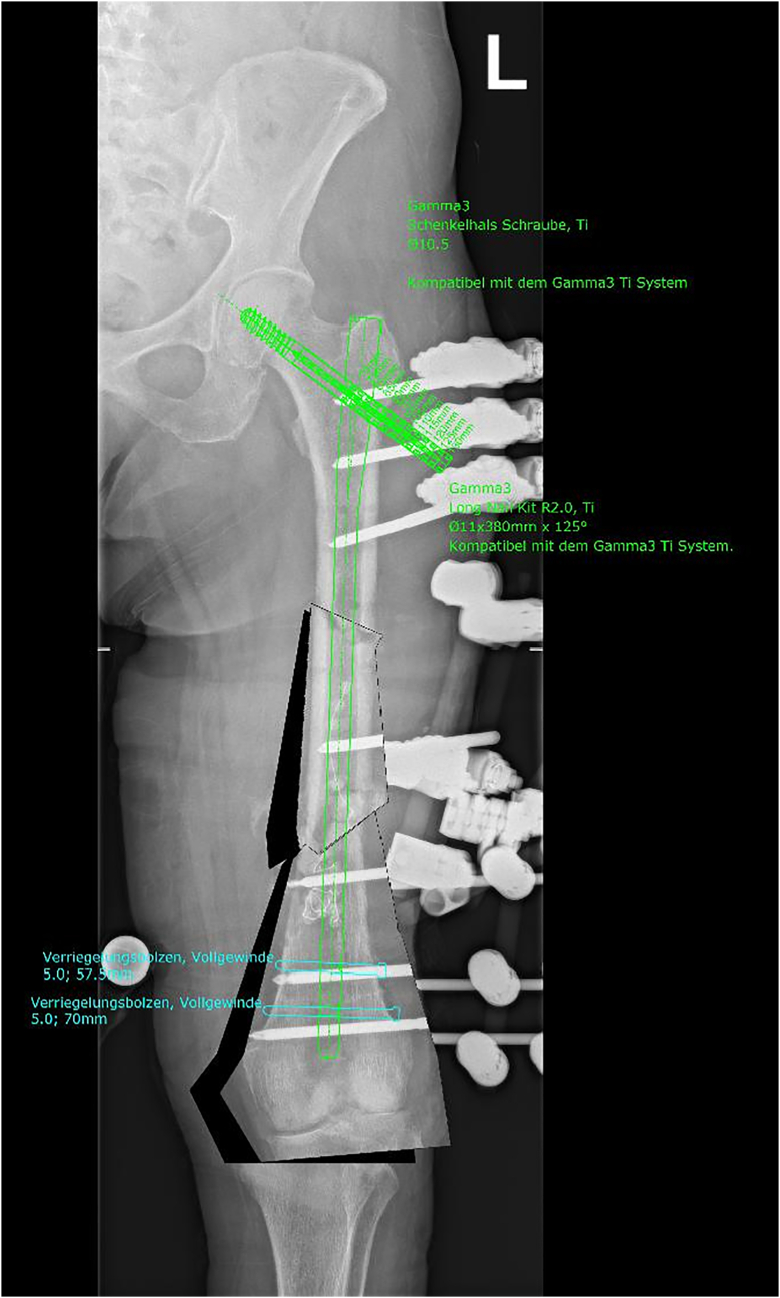
X-ray with software-based pre-operative planning with long gamma nail with cephalo-medullary screw and double distal locking.

**Fig. 5:**
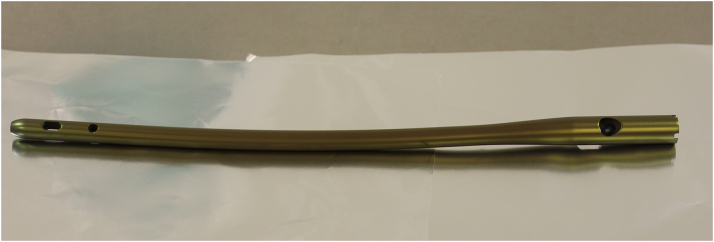
Gamma-nail with low-amount silver coating before implantation.

**Fig. 6:**
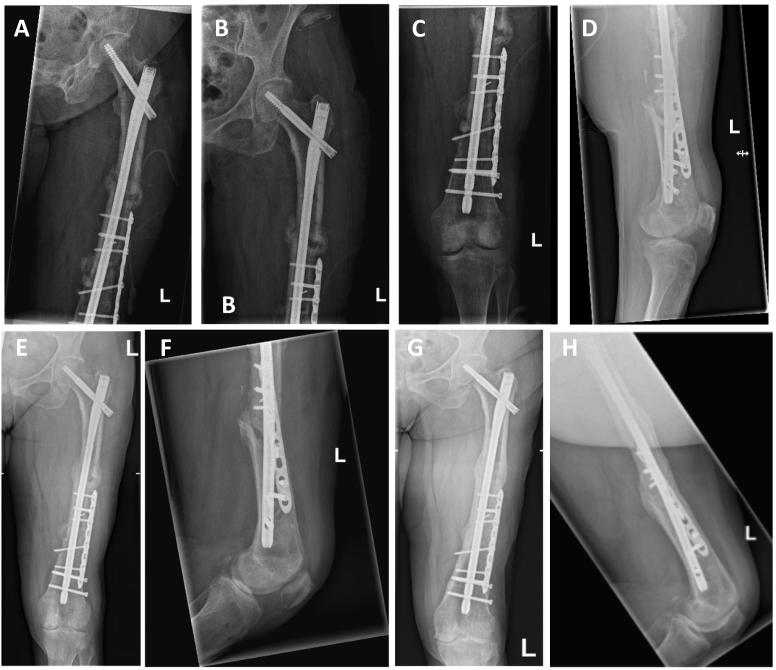
Postoperative ap (A + B) and lateral (C + D) X-rays after removal of the antibiotic beads, re-debridement and re-osteosynthesis with silver-coated gamma nail and augmentation plate. X-rays at 3 weeks after re-osteosynthesis (E + F). X-rays at 26 months after re-osteosynthesis with full consolidation of the proximal fracture and the distal non-union site without implant failure (G + H).

Preoperative software-based planning revealed a 370 mm long and 11 mm thick gamma nail (Stryker, Duisburg, Germany) to be suitable for re-osteosynthesis both for stabilization of the fresh fracture of the proximal and of the infected non-union site at the distal part of the femoral shaft (Fig. 4). A gamma nail with the pre-determined geometry was then transferred from the hospital for low amount of silver coating to the coating company (HyProtect®, Bio-Gate, Nürnberg, Germany). Upon silver coating and gamma-sterilization, the nail was received back two weeks later (Fig. 5). After another week, soft tissue conditions and CRP value normalized and made bone reconstruction possible. For this, the monolateral fixateur was removed as well as the antibiotic beads and the antibiotic loaded PMMA bone cement. There were no signs of infection neither in the soft tissue nor in the bone. The silver-coated nail then was implanted via a trochanteric approach after new adequate debridement of the non-union site. Upon implantation of the nail, there was a remaining instability of the non-union at the distal femoral shaft due to the 3 cm circumferential defect. Therefore, an additional 8-hole locking augmentation plate was placed at the lateral aspect of the distal femoral shaft, which resulted in sufficient stability. The defect itself was filled with 10 ml of a calciumsulfate-hydroxyapatite bone substitute material loaded with gentamicin (Cerament G®, Bonesupport, Lund, Sweden) and primary wound closure was performed. Partial weight-bearing for 6 weeks war performed followed by full weight bearing. Systemic antibiotic therapy was changed after three weeks to Cotrimoxazol (960 mg 2 × 1) for another three weeks. Postoperative X-rays showed a good alignment of the fracture and non-union site with correct positioning of the intramedullary nail and of the augmentation plate (Fig. 6A–D). The wounds healed without any further complication within three weeks and the patient could be discharged.

The patient showed successful fracture healing of the proximal fracture site within six weeks (Fig. 6E + F) and did not show any signs of recurrence of infection. Successful bone healing of the former infected nonunion was also achieved. After a follow up of 26 months, X-rays showed stable consolidation of both the fracture and of the previously infected non-union site (Fig. 6H + G). No further surgical intervention or antibiotic treatment had to be carried out in the meantime. No silver associated adverse events, such as argyria with blue-grey dyscolorization of the skin, were seen either.

## Discussion

To our best knowledge, this is the first case to report about low-amount silver coating of an intramedullary nail for the treatment of an infected nonunion. It was previously reported for successful management with silver-coated knee arthrodesis implants for a recurrent periprosthetic knee infection [6] and a complex septic knee arthritis case caused by *Aspergillus fumimatus* [7]. The low-amount silver coating is not officially approved yet and specific informed consent must be obtained from the patient before its use. The silver coating can be applied to all types of metals, such as titanium [6], [7] and stainless steel [8] as well as polymers, which enables the individual choice of the implant for the treatment of complex cases possible for the surgeon.

The infected non-union in this case was complicated by an additional fresh fracture due to a fall of the patient during the 2-stage treatment of the infected non-union that was finally treated with a silver-coated gamma nail. This silver coating technology uses only a relatively low amount of silver, which makes silver specific adverse events, such as argyria with dyscolorization of the skin around the indwelling silver-coated implant as reported for high-amount silver coating technologies [1], [9] very unlikely. The two previously reported cases did not show any silver-adverse events either [6], [7]. The mode of action of the low-amount silver coating technology is the release of silver ions from a 10 to 30 nm thin siloxane layer that is directly put on the implant surface [8]. The broad antimicrobial effect of silver was a clear benefit in the presented case with a polymicrobial infection as silver is known to be effective against different gram-positive strains, such as *S. aureus* and *S. epidermidis,* as well against gram-negative strains, such as *E. coli* [4].

The released silver ions did not influence successful bone healing neither at the fracture site nor at the former infected nonunion site. This suggests that the low-amount silver coating technology does not negatively interfere with fracture or non-union healing.

## Conclusions

This case illustrates the following learning points:

1. individual low-amount silver coating of intramedullary nails is possible and can be used in complex bone infections surgery.
2. Antimicrobial aspects of the released silver ions showed to be beneficial in the presented case to avoid recurrence of infection in the presented case
3. Low-amount silver coating technology did not interfere with fracture or non-union bone healing in this case.

## Declaration of competing interest

Volker Alt is consultant for Bio-Gate AG, Nürnberg, Germany.
